# Supplementary material for: A catalog of small proteins from the global microbiome
Source: Nat Commun. 2024 Aug 31;15:7563. doi: 10.1038/s41467-024-51894-6 (PMC11364881; doi:10.1038/s41467-024-51894-6)
Supplement: Supplementary file 1 — Supplementary Information [file 41467_2024_51894_MOESM1_ESM.pdf]

Supplemental Figures

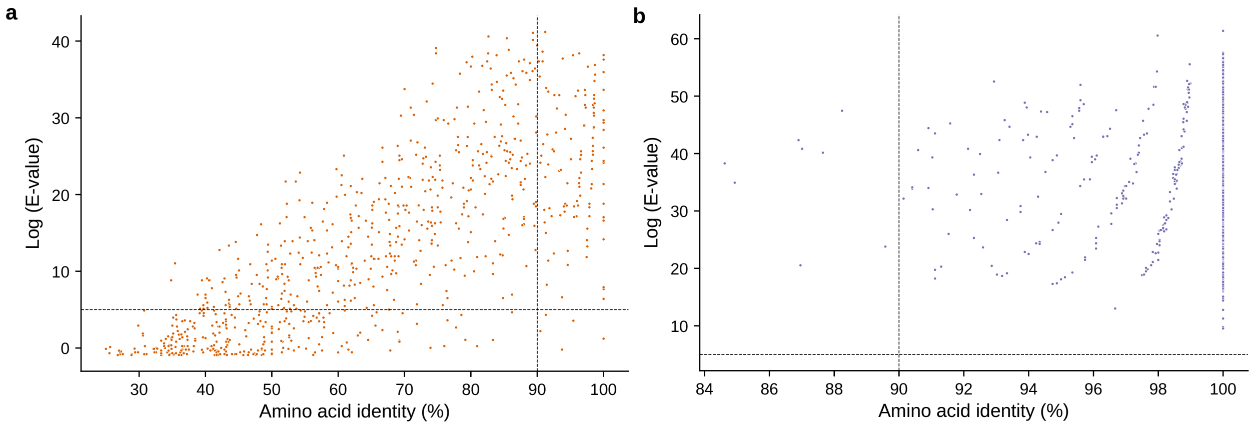

Supplementary Fig. 1 Clusters significance validation (a) We aligned 1,000 randomly selected singleton sequences against the representative sequences of non-singleton clusters using SWIPE (an exhaustive search method). Only 44 (4.4%) of the sequences with E-value ≤10^{-5}, identity ≥ 90%, and coverage ≥ 90% were considered significant and represent false negatives from the heuristic alignment method used. **(b)** We aligned 1,000 randomly selected sequences against the representative sequences of the clusters they belong to using SWIPE. 99.2% (992 / 1,000) of the alignments were significant (E-value ≤ 10^{-5}, identity ≥ 90%, and coverage ≥ 90%).

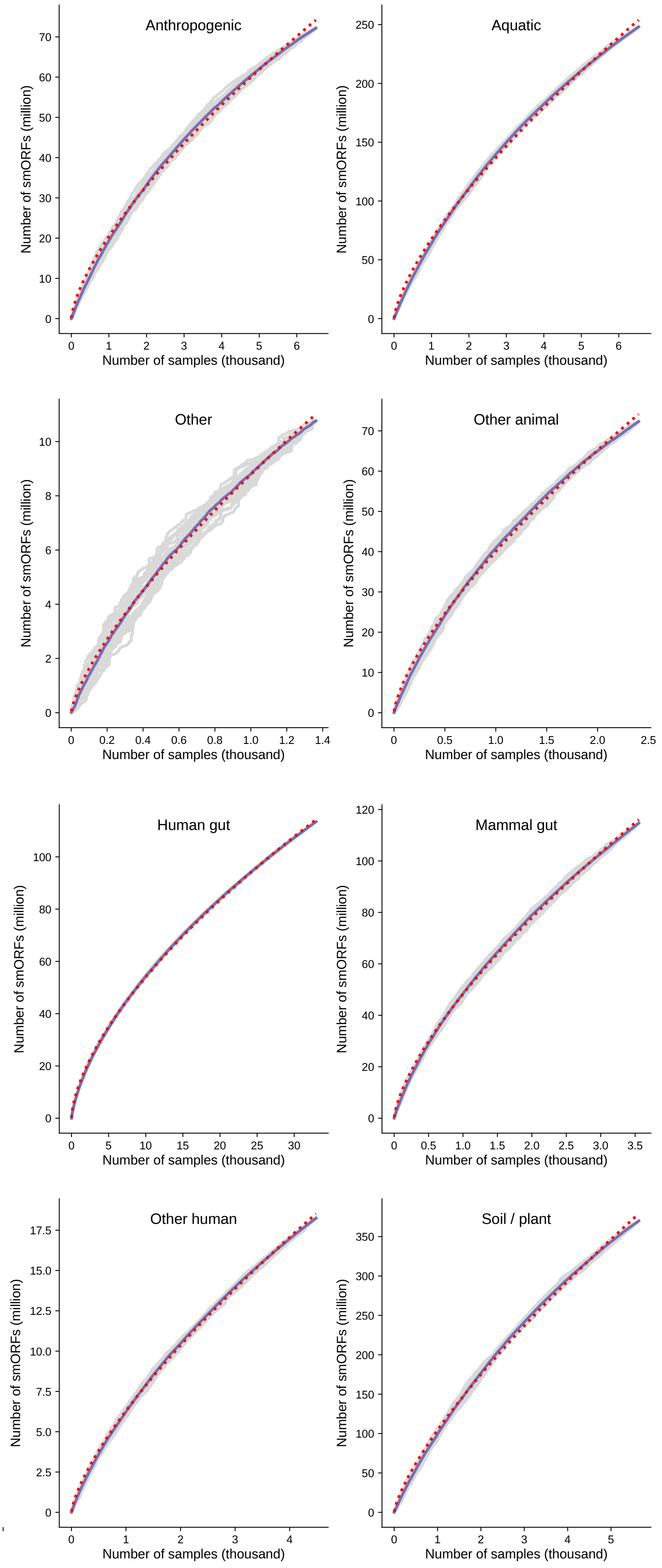

Supplementary Fig. 2 The smORF accumulation curves across habitats (a) The grey lines represent the 24 permutations of sample selection for each broad habitat category. The blue line is the average of these permutations. The red line is the fit of Heap's Law ($N=k \cdot sample^{\alpha}$). It indicates that the amount of smORFs in any habitat is not saturated.

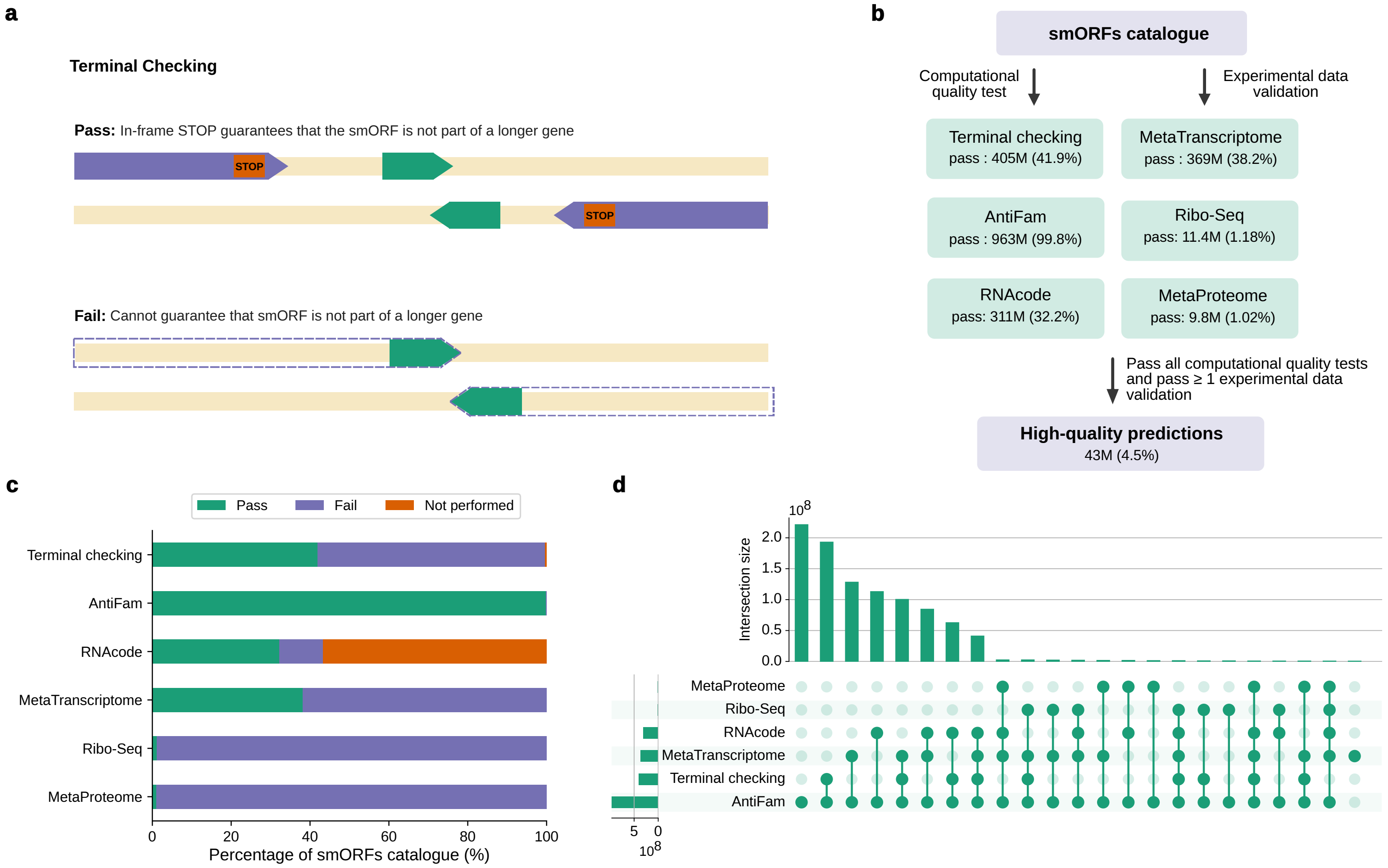

Supplementary Fig. 3 Quality assessment workflow and overlap (a) To rule out the possibility that a smORF is part of a longer gene due to contig fragmentation, we searched for an in-frame STOP codon upstream of the smORF START. **(b)** The computational quality tests include (i) Terminal checking to reduce the risk that the smORF is derived from a fragmented longer gene (as illustrated in **a**); (ii) AntiFam searches to avoid spurious protein families; and (iii) RNAcode estimated coding potential. The experimental data validation consists of mapping the metatranscriptomic and Ribo-Seq reads downloaded from the public database and exactly matching metaproteomic peptides downloaded from the Proteomics Identification Database (PRIDE). SmORFs were considered high-quality predictions if they passed all computational quality tests and were found in at least one experimental dataset. **(c)** Fraction of GMSC smORFs for each test. RNAcode was performed only on clusters with at least 8 members. Terminal checking was performed only on smORFs derived from metagenomes. **(d)** The upset plot shows the number of overlapping sequences passing each quality testing method.

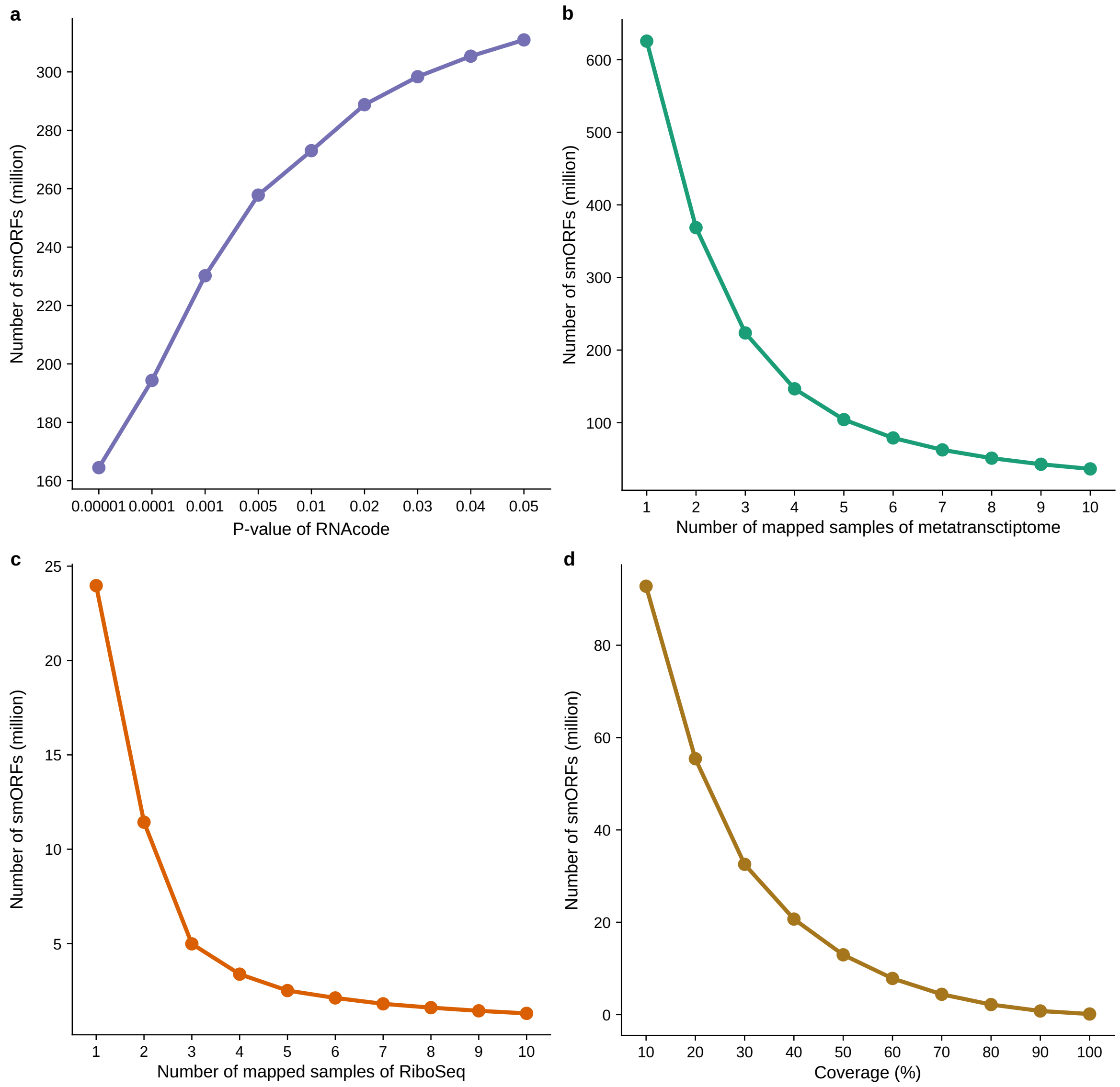

Supplementary Fig. 4 Effect of different thresholds on quality control (a) The number of smORFs with high coding potential as estimated by RNAcode, using different P-value thresholds. **(b)** The number of smORFs with transcriptional evidence, using different thresholds for the minimal number of samples required for detection. **(c)** The number of smORFs with translational evidence, using different thresholds for the minimal number of samples required for detection. **(d)** The number of detected smORFs in metaproteomics data, using different thresholds for the required k-mer coverage of each smORF-encoded small protein (**Methods**).

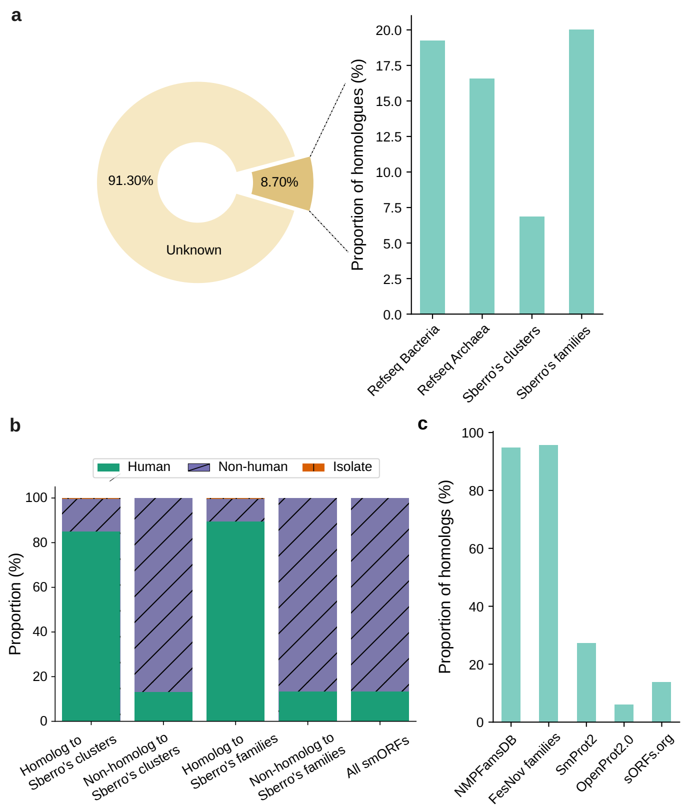

Supplementary Fig. 5 Comparison of reference small protein datasets (a) Shown is the fraction of smORFs from high-quality predictions that are homologous to reference small protein datasets. **(b)** The comparison of the proportions of smORFs from human or non-human habitats between homologs or non-homologs to small protein clusters and conserved families from the Sberro human microbiome dataset. **(c)** Shown is the fraction of GMSC smORFs that are homologous to NMPfamsDB, FesNov families, smProt2, OpenProt2.0, and sORF.org.

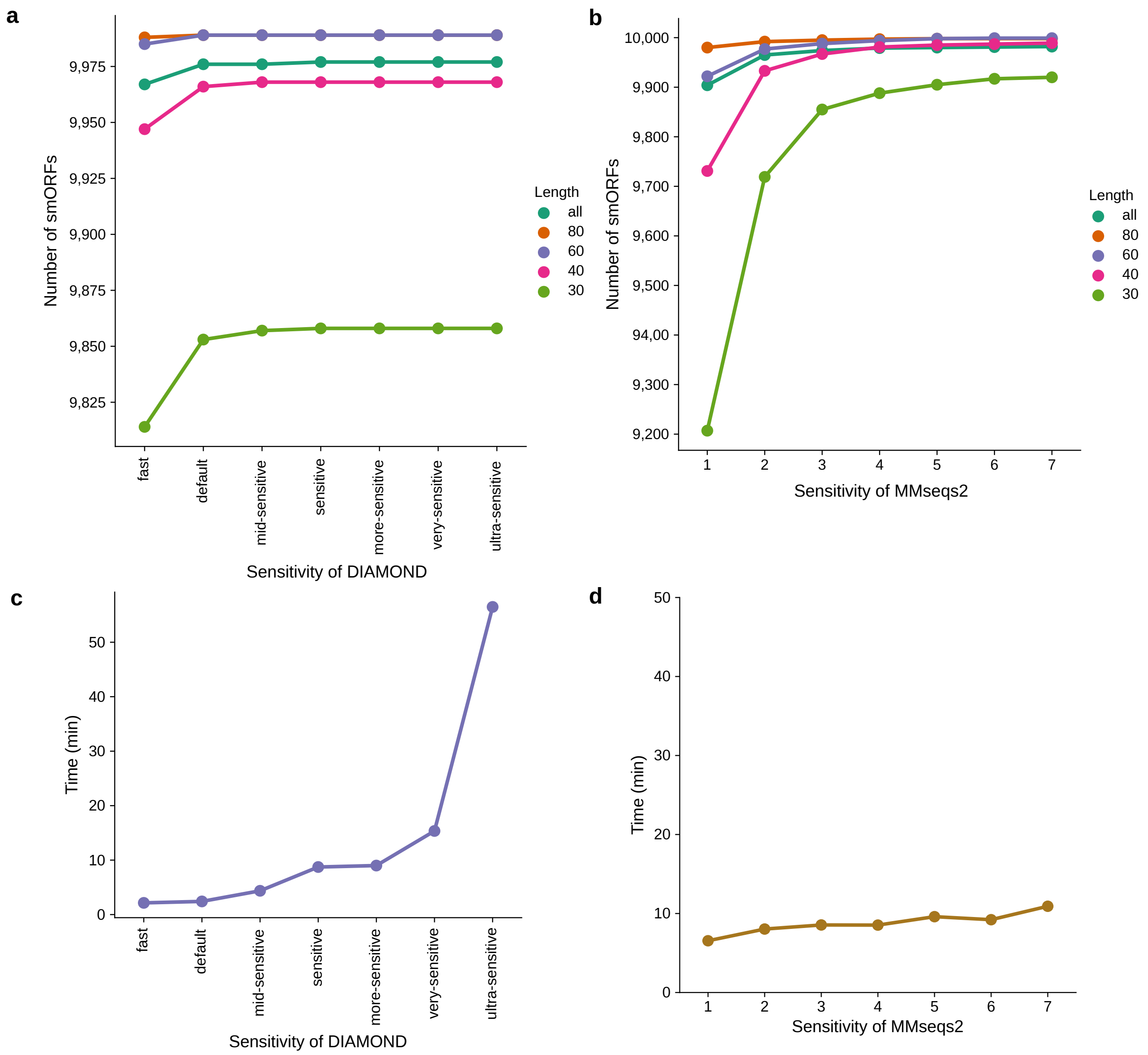

Supplementary Fig. 6 Benchmark of sensitivity modes between DIAMOND and MMseqs2 (a) Shown is the recovery amount of 10,000 randomly selected smORFs with different lengths under different sensitivity modes by DIAMOND. **(b)** Shown is the recovery amount of 10,000 randomly selected smORFs with different lengths under different sensitivity parameters by MMseqs2. **(c)** Shown is the time cost for DIAMOND to map 10,000 randomly selected smORFs to the smORF family representatives under different sensitivity modes. **(d)** Shown is the time cost for MMseqs2 to map 10,000 randomly selected smORFs to the smORF family representatives under different sensitivity parameters.
