# Supplementary material for: A catalog of small proteins from the global microbiome
Source: Nat Commun. 2024 Aug 31;15:7563. doi: 10.1038/s41467-024-51894-6 (PMC11364881; doi:10.1038/s41467-024-51894-6)
Supplement: Supplementary file 3 — Description of Additional Supplementary Files [file 41467_2024_51894_MOESM3_ESM.pdf]

# Description of Additional Supplementary Files

## File Name: Supplementary Data 1

## Description:

**Metadata of metagenomes and isolated genomes** Each metagenomic sample is provided with project accession, publication, habitat including environment and host, geographic coordinates, and collection dates. Basic assembly information for each sample includes the number of assembled base pairs (bps) and the assembly N50. The number of non-redundant smORFs catalogue and the number of smORF families for each metagenomic sample and the isolated genomes from the Progenomes2 database are shown.

## File Name: Supplementary Data 2

## Description:

**The distribution of smORFs in the broad habitat categories** Displayed the various habitats included in each broad habitat category, number of samples, number of redundant smORFs, number of non-redundant smORFs catalogue, and number of smORF families.

## File Name: Supplementary Data 3

## Description:

**Metatranscriptomic, Ribo-Seq, and metaproteomic datasets used in the experimental validation** We downloaded 221 publicly available metatranscriptomic datasets, which are paired with the metagenomic samples that we used in the catalogue. We downloaded 142 publicly available Ribo-Seq datasets. We downloaded peptide datasets of 108 metaproteome projects from the Proteomics Identification Database (PRIDE). The accession and source of the projects are listed.

## File Name: Supplementary Data 4

## Description:

**Conserved domain annotation of the catalogue** The number of annotated sequences and detailed descriptions for each conserved domain are provided. Pfam accessions are grouped by Pfam clan and short description.

## File Name: Supplementary Data 5

## Description:

**Conserved domain annotation of the small protein families present in multiple phyla and habitats** The number of members of the small protein families, the number of species

of the small protein families, and conserved domain annotation with description are provided.

## File Name: Supplementary Data 6

## Description:

**smORF density across different phyla** The number of redundant smORFs and the assembled base pairs are shown and used to calculate the smORF density for each phylum.
